# Gastric cancer and the TNT era: have we solved the timing question, or only redistributed the treatment?

**DOI:** 10.3389/fonc.2026.1949844

**Published:** 2026-09-15

**Authors:** Serdar Gumus, Ibrahim Cogal, Ishak Aydın, Cem Kaan Parsak

**Affiliations:** Department of Surgical Oncology, Çukurova University, Adana, Türkiye

**Keywords:** gastric cancer, neoadjuvant chemotherapy, perioperative chemotherapy, total neoadjuvant therapy (TNT), treatment timing

## Abstract

Perioperative chemotherapy has become standard of care for locally advanced resectable gastric cancer, supported by landmark trials including MAGIC, FLOT4, PRODIGY, RESOLVE, and RESONANCE. However, a methodological question remains insufficiently addressed: to what extent can the observed benefits of perioperative treatment be attributed to an independent effect of treatment timing rather than to the consequences of delivering systemic therapy before surgery? Western and East Asian research traditions have approached this question differently. Western trials primarily tested whether adding a neoadjuvant component improves outcomes, whereas East Asian trials established the value of adjuvant therapy after high-quality D2 surgery. Consequently, most landmark trials were not designed to isolate timing as an independent variable, and even same-regimen comparisons may be influenced by differences in treatment completion, cumulative exposure, and patient selection. Despite this uncertainty, the field has shifted toward total neoadjuvant therapy (TNT), redistributing more systemic treatment to the preoperative period. Depending on the protocol, TNT may represent treatment redistribution, intensification, or both. Although randomized trials have evaluated modified preoperative strategies, confirmatory phase III evidence specifically isolating treatment timing under comparable systemic treatment exposure remains lacking, leaving the independent oncological value of treatment sequence uncertain. Future trials should directly compare perioperative and postoperative delivery of the same systemic regimen under comparable planned cumulative treatment exposure while incorporating molecular stratification. Rather than assuming earlier systemic treatment is superior, studies should determine whether it provides an independent oncological advantage beyond improved treatment completion and patient selection, and whether this effect varies across biological subgroups.

## Introduction

Despite continued refinement of surgical technique, relapse after resection alone remains common in locally advanced gastric cancer, which has driven two decades of effort to integrate systemic therapy into the surgical pathway (1–8). This effort has clearly established that adding systemic therapy improves outcomes relative to surgery alone in several landmark trials. What is far less settled, and has received comparatively little explicit methodological scrutiny, is a distinct question: not whether systemic therapy should be given, but when, within the overall treatment course, it should be delivered. Because most pivotal trials in this field were designed to test the former question, their results are frequently interpreted as if they had also answered the latter.

By an independent timing effect, we refer specifically to a survival or recurrence benefit attributable to *when* an otherwise equivalent systemic treatment is delivered relative to surgery—under comparable planned cumulative treatment exposure—rather than to the several downstream clinical consequences that earlier delivery may also produce, such as improved treatment completion, tumor downstaging and higher R0 resection rates, or the preoperative selection of patients according to tumor biology. These downstream consequences are clinically meaningful and may themselves support a preoperative strategy in practice, but they are not, on their own, evidence of a distinct biological timing effect. A trial designed to isolate timing should therefore maintain comparable planned cumulative treatment exposure across arms and account for differential patient selection, ideally through randomization and an intention-to-treat framework that includes patients who progress, discontinue treatment, or do not proceed to surgery during preoperative therapy. Such a design would allow the effect of earlier systemic exposure to be more clearly distinguished from the correlated clinical consequences of preoperative treatment sequencing.

Historically, the Western approach primarily sought to answer the question of whether outcomes improve if systemic therapy is administered before surgery. In contrast, the East Asian paradigm focused on how much additional benefit systemic therapy provides after optimal, especially D2, surgery. These are not alternative approaches but methodologically distinct conceptual frameworks. While Western studies investigated whether moving treatment to the neoadjuvant setting improved survival, Eastern studies questioned whether adjuvant therapy remained clinically necessary after high-quality surgery.

## Two paradigms, one unanswered question

As a consequence of this distinction, the landmark MAGIC trial compared perioperative ECF with surgery alone (1). Therefore, the study tested not the timing of systemic therapy, but rather the addition of systemic treatment itself. Likewise, FLOT4—which established the current Western standard—compared perioperative FLOT with perioperative ECF/ECX and demonstrated superiority of FLOT; however, it did not evaluate whether the same effective regimen administered entirely in the postoperative setting would yield similar outcomes (2).

Conversely, Eastern practice was shaped by ACTS-GC and CLASSIC, which demonstrated survival benefits from adding adjuvant S-1 and CAPOX following high-quality D2 surgery, but did not question whether neoadjuvant treatment was essential (7, 8).

## Landmark trials revisited: addition versus timing

More recently, the PRODIGY and RESOLVE trials revisited the role of the neoadjuvant component and reopened discussion regarding perioperative treatment in East Asia (3–5). In PRODIGY, patients with resectable locally advanced gastric cancer were randomized to neoadjuvant DOS followed by D2 surgery and adjuvant S-1, or upfront D2 surgery followed by adjuvant S-1. The initial analysis demonstrated a progression-free survival benefit, and subsequent long-term follow-up confirmed an overall survival advantage with the addition of neoadjuvant DOS. However, PRODIGY did not redistribute an otherwise equivalent systemic regimen around surgery; rather, three cycles of a multidrug DOS regimen were added before surgery to an adjuvant S-1 backbone shared by both groups. Consequently, the observed benefit cannot be attributed specifically to treatment timing, because earlier systemic exposure, greater cumulative treatment burden, and regimen intensification were introduced simultaneously (3, 4).

RESOLVE randomized patients to three treatment strategies: perioperative SOX, adjuvant SOX, and adjuvant CAPOX. In the final analysis, 5-year overall survival was 60.0% with perioperative SOX, 61.0% with adjuvant SOX, and 52.1% with adjuvant CAPOX. Both SOX-based strategies demonstrated favorable overall survival compared with adjuvant CAPOX. Although the 5-year overall survival estimates were numerically similar between perioperative and adjuvant SOX, RESOLVE was not designed to establish equivalence between these two strategies; therefore, their similar survival estimates should not be interpreted as evidence of equivalent efficacy. Nevertheless, these findings raise an important methodological question: to what extent do the favorable outcomes observed with the SOX-based strategies reflect the regimen itself rather than the timing of its delivery?. RESOLVE nevertheless was not a completely exposure-matched timing comparison. Both SOX strategies included eight planned SOX cycles—three preoperative and five postoperative cycles in the perioperative arm versus eight postoperative cycles in the adjuvant arm—but the perioperative strategy was followed by three cycles of S-1 monotherapy. Therefore, although RESOLVE closely approaches the timing question, the treatment schedules were not entirely identical beyond the timing of SOX administration (5).

One of the most illustrative examples of this shift was the RESONANCE trial, which randomized patients with clinical stage II–III resectable gastric adenocarcinoma to perioperative SOX or surgery followed by adjuvant SOX (6). The trial demonstrated a disease-free survival advantage favoring the perioperative approach, with 3-year disease-free survival rates of 61.7% versus 53.8% (HR 0.76, p=0.019). However, this benefit was not uniform across disease stages: subgroup analysis demonstrated a significant disease-free survival benefit in stage III but not stage II disease, suggesting that the value of earlier systemic treatment may depend substantially on patient selection. Moreover, completion of all eight planned cycles differed markedly between the perioperative and adjuvant groups (41.1% vs. 19.2%), making it difficult to separate the effect of earlier systemic exposure from the clinical advantage of improved treatment delivery. Thus, RESONANCE provides important randomized evidence supporting perioperative SOX, particularly in higher-stage disease, but does not establish a universal or purely biological timing advantage. Rather, its findings suggest that disease stage and treatment deliverability may modify the value of treatment timing (6).

More recently, the phase III ASTRUM-006 trial demonstrated an event-free survival benefit with perioperative serplulimab-based therapy in patients with PD-L1-positive resectable gastric or gastro-esophageal junction adenocarcinoma. However, ASTRUM-006 does not isolate the effect of treatment timing because the experimental and control strategies differed in more than the timing of systemic therapy, including the incorporation of immunotherapy and differences in postoperative treatment. Thus, although ASTRUM-006 provides important evidence supporting perioperative chemoimmunotherapy in a biomarker-selected population, its observed benefit cannot be attributed specifically to treatment timing (9).

Accordingly, one critical question remains unresolved: when the systemic regimen and planned cumulative treatment exposure are comparable, does perioperative therapy provide an independent oncological advantage over postoperative therapy alone, beyond the effects of improved treatment completion and patient selection?

## Real-World evidence: a heterogeneous landscape

Uncertainty regarding optimal timing is not limited to randomized trials; real-world evidence remains similarly heterogeneous. Initial real-world signals appeared to favor perioperative treatment. Eom et al. reported improved long-term outcomes in a Korean propensity-score–matched study, increasing interest in neoadjuvant approaches in Asia (10). Similarly, Wang et al. reported improved overall survival with neoadjuvant strategies using Chinese National Cancer Center data (11). However, these retrospective studies did not isolate timing effects and remained vulnerable to treatment selection bias, treatment tolerance, and unmeasured biological factors.

More recent analyses have produced mixed findings. Su et al. reported improved R0 resection rates and survival outcomes with perioperative therapy but could not disentangle timing from treatment completion and patient selection effects (12). In contrast, Elangovan et al. found no significant advantage in overall or recurrence-free survival using propensity-matched analysis (13).

Taken together, these studies suggest an intriguing pattern: perioperative treatment appears associated with better pathological response and, in some series, improved recurrence control, yet its translation into consistent long-term survival benefits remains uncertain. Importantly, perioperative cohorts inherently consist of a biologically selected population—patients who do not progress during neoadjuvant therapy, successfully undergo surgery, and complete treatment. Thus, it remains unclear how much of the observed benefit derives from treatment timing versus hidden biological selection. This heterogeneous real-world landscape suggests that not only the necessity of systemic therapy but also its optimal timing remains incompletely defined.

## The rise of total neoadjuvant therapy: has treatment timing moved ahead of the evidence?

It is important to distinguish between two conceptually different strategies subsumed under the TNT label. In some protocols, the total planned number of cycles and cumulative dose remain unchanged, and only their sequence relative to surgery is altered—systemic therapy is redistributed rather than intensified. In others, additional cycles are incorporated into the preoperative period on top of what would otherwise have been delivered, representing genuine treatment intensification. These two strategies carry different rationales and different evidentiary requirements: redistribution primarily tests a timing hypothesis, whereas intensification introduces an additional dose–response question that cannot be attributed to timing alone. The TNT literature reviewed below spans both categories, and this distinction should be kept in mind when interpreting the reported outcomes.

The CRITICS-II study moved beyond the classic perioperative model by evaluating three entirely preoperative treatment strategies—chemotherapy alone, chemotherapy followed by chemoradiotherapy, and chemoradiotherapy alone—while omitting postoperative therapy. One-year event-free survival was 68%, 84%, and 78%, respectively, while pathological complete response rates were 8%, 20%, and 13%. Preoperative chemotherapy alone failed the prespecified EFS activity threshold, whereas both chemoradiotherapy-containing strategies met it; considering survival, toxicity, postoperative outcomes, and pathological response, chemotherapy followed by chemoradiotherapy emerged as the preferred strategy for further investigation. However, CRITICS-II was a randomized phase II selection trial designed to identify the most promising preoperative strategy for subsequent phase III evaluation rather than to establish definitive long-term survival superiority or isolate treatment timing as an independent variable (14).

The phase III PREACT trial provides further insight into the relationship between preoperative treatment intensification and long-term outcomes. Compared with preoperative SOX chemotherapy alone, the addition of preoperative chemoradiotherapy increased the pathological complete response rate (12.0% vs. 2.1%) and R0 resection rate (81.0% vs. 74.5%). However, these improvements did not translate into differences in 3-year disease-free survival (53.6% vs. 53.9%; HR 1.02) or overall survival (62.8% vs. 60.5%; HR 0.97). Importantly, the trial was terminated prematurely because of slow accrual, enrolling only 204 of the planned 682 patients, which limits definitive conclusions regarding survival. Nevertheless, PREACT illustrates that improvements in pathological response and resectability do not necessarily translate into improved long-term oncological outcomes (15).

Similarly, early-phase studies by Badgwell et al., retrospective analyses from Yang et al. at Memorial Sloan Kettering Cancer Center, and recent total neoadjuvant FLOT (FLOT×8) experiences reported encouraging findings regarding treatment delivery, completion, and pathological response; however, they have not yet established an improvement in long-term overall or disease-free survival (16–18).

Notably, Yang et al. found that patients receiving TNT completed a greater proportion of cycles containing all planned chemotherapy agents, but the overall proportion completing all planned cycles did not differ significantly, and no improvement in recurrence-free or overall survival was demonstrated (17). Likewise, FLOT×8 was associated with higher treatment completion and a numerically higher, but not statistically significant, pathological complete response rate, without a demonstrated survival advantage (18).

It is important to note the level of evidence underlying these observations. Badgwell et al. reported a single-arm phase 1 feasibility study; the Yang et al. experience is a retrospective single-center comparison; and the FLOT×8 experience is a propensity-matched retrospective cohort rather than a randomized trial (16–18). Importantly, despite the growing randomized evidence evaluating modified or intensified preoperative strategies, no completed phase III trial has specifically isolated treatment timing by comparing otherwise equivalent systemic regimens delivered at different points relative to surgery under comparable planned cumulative treatment exposure. This evidentiary gap is central to this Perspective: the redistribution of systemic therapy toward the preoperative period is increasingly entering clinical discussion and, in some centers, practice, despite the absence of confirmatory evidence establishing an independent oncological benefit of treatment timing.

This raises an important methodological question once again: before establishing the long-term oncological value of earlier systemic exposure across molecular subgroups, how rational is it to move an increasing proportion—or, in some protocols, the entirety—of systemic therapy into the preoperative period? TNT in gastric cancer should therefore not be regarded as a uniform strategy: depending on the protocol, it may represent treatment redistribution, true intensification, or a combination of both. These distinctions are essential when interpreting whether observed benefits arise from treatment timing, cumulative treatment exposure, or both.

## Discussion

Perhaps the more important question is not how much systemic therapy can be moved into the neoadjuvant setting, but whether it is being delivered at the biologically optimal time. Current literature strongly supports the necessity of systemic therapy. However, it remains difficult to conclude that the ideal timing of treatment has been definitively established across all regimens and biological subgroups.

We therefore believe that the next major phase III trial in gastric cancer should directly address the timing hypothesis by comparing perioperative and postoperative delivery of the same effective systemic treatment under comparable planned cumulative treatment exposure, while incorporating molecular biological subtypes—such as microsatellite instability (MSI) status, Epstein–Barr virus positivity, and Lauren histological classification—to determine whether the optimal timing of systemic therapy varies according to tumor biology.

This is not a purely theoretical consideration: an exploratory analysis of the MAGIC trial found that patients with MSI-high or mismatch repair-deficient tumors treated with surgery alone had better survival than those who received perioperative chemotherapy, whereas microsatellite-stable patients benefited from chemotherapy as expected (19). If systemic therapy itself can be neutral or even detrimental in specific molecular subgroups, it is plausible that the optimal timing of that therapy—not only its use—may also vary by subgroup. Stratifying future trials by molecular subtype would therefore help clarify whether the biological benefit of earlier systemic exposure is uniform across gastric cancer subtypes or restricted to specific tumor biologies, an aspect that remains unaddressed by the current evidence base.

## Data Availability

The original contributions presented in the study are included in the article/supplementary material. Further inquiries can be directed to the corresponding author.
